# Comparative Study on the Physical and Chemical Properties Influenced by Variations in Fermentation Bacteria Groups: Inoculating Different Fermented Mare’s Milk into Cow’s Milk

**DOI:** 10.3390/foods14081328

**Published:** 2025-04-11

**Authors:** Fanyu Kong, Qing Zhao, Shengyuan Wang, Guangqing Mu, Xiaomeng Wu

**Affiliations:** School of Food Science and Technology, Dalian Polytechnic University, Dalian 116034, China

**Keywords:** back-slopping, microbiota diversity, strain transmission, association analysis

## Abstract

Fermented strains play a crucial role in shaping the physicochemical properties and functionality of fermented cow’s milk. The natural fermentation system demonstrates a certain degree of stability and safety after undergoing continuous domestication. Fermented mare’s milk has been consumed for its intestinal health benefits in regions such as Xinjiang and Inner Mongolia in China. This consumption is closely related to the fermented strains present. Consequently, from the perspective of fermented strains, this study aimed to compare the microbiota diversity of naturally fermented mare’s milk with that of inoculated fermented cow’s milk, using it as a fermentation system to develop new functional fermented cow’s milk products. Water retention, rheology, texture, pH, and titration acidity were analyzed to evaluate the quality of fermented cow’s milk with the obtained transmission strain system. Importantly, the correlation between the property of fermented cow’s milk and the diversity of fermentation system has been thoroughly analyzed. The findings indicate that the gel property of fermented cow’s milk is not directly linked to the strain diversity or the core strain of fermentation. Instead, the abundance of *Lactobacillus*, *Lactococcus*, *Hafnia-Obesumbacterium*, *Leuconostoc*, *Acetobacter*, and *Acinetobacter* bacteria significantly influences the quality of fermented cow’s milk. Consequently, this study has successfully developed a new type of fermented cow’s milk and provided a reliable theoretical foundation for the functional enhancement of specialized fermented cow’s milk products.

## 1. Introduction

Northern regions of China, including Inner Mongolia and Xinjiang, are home to numerous natural, traditionally fermented dairy products [1]. Generally, traditional fermented dairy products are typically crafted domestically or in small-scale settings through natural fermentation processes. This fermentation arises from inherent activities of microbiota in raw milk, production environments, and equipment. Product attributes depend on variables like milk quality, processing techniques, and regional or local climatic conditions [2]. Over time, this method results in minimal changes to the taste and flavor of the product, and even maintains a stable fermentation culture. Fermented mare’s milk is a highly nutritious dairy product rich in vitamins and minerals such as phosphorus and calcium, as well as essential fatty acids like linoleic acid and linolenic acid [3]. These components positively impact the immune system, help maintain stable blood pressure, and contribute to the health of the kidneys, endocrine glands, intestinal system, liver, nervous system, and vascular system [4]. The substances that produce functional activity in fermented mare’s milk may come from peptides during the fermentation process and inherent microbiota. The microbiota includes *Lactobacillus*, *Lactococcus*, *Acetobacter*, *Streptococcus*, *Serratia*, and *Leuconostoc* bacteria, as well as *Kazachstania*, *Kluyveromyces*, *Trichosporonaceae*, *Pichia*, and others [5]. The relative abundance of the anti-inflammatory *Clostridium prausnitzii* in the gut has been observed to increase after consuming fermented mare’s milk [3]. Fermented mare’s milk can also enhance intestinal barrier function: the *Lactobacillus* it contains can promote the production of tight junction protein in intestinal epithelial cells and reduce inflammatory stimulation [6]. However, due to the scarcity of mare’s milk, fermented mare’s milk is not commonly used as a consumer product among life’s dairy offerings. At present, the acquisition of fermented mare’s milk on the market is more difficult, the product variety is limited, and the price is expensive, almost 10 times more than the price of the same amount of easily obtained fermented cow’s milk.

As the concept of total health gains popularity, fermented cow’s milk products, which contain probiotics such as *Lactobacillus* that promote digestion and regulate intestinal microbiota, have become favored by consumers for their numerous health benefits [7]. Fermented cow’s milk is produced through the fermentation process involving *Lactobacillus delbrueckii* subsp*. bulgaricus* and *Streptococcus thermophilus*, and it offers a range of benefits. Regarding intestinal health, research has indicated that the probiotics present in fermented cow’s milk can regulate the intestinal microbiota and boost the population of beneficial bacteria, such as *Bifidobacterium*. These beneficial bacteria produce short-chain fatty acids, which are advantageous for intestinal health and immunity [8]. Due to the simplicity of the strains, researchers develop and add functional probiotics, such as *Limosilactobacillus fermentum*, *Lactiplantibacillus plantarum*, and *Bifidobacterium animalis* subsp*. lactis*, to create a new robust fermentation system for producing fermented cow’s milk [9]. The inclusion of these probiotics not only enhances the flavor of the fermented cow’s milk but also imparts new functionalities, such as alleviating constipation, treating diarrhea, and addressing enteritis [9]. In addition, compared to cow’s milk, the microbiota in fermented cow’s milk can reduce the content of high-molecular-weight proteins and promote digestion and absorption in the body [10]. Fermented cow’s milk is rich in calcium, and fermentation may enhance the bioavailability of this mineral, as well as vitamins B12, B2, and B5, among others. In terms of health benefits beyond gut health, fermented cow’s milk can boost the immune system by increasing the production of immunoglobulin A [11]. However, most of these products contain stabilizers and thickeners to enhance their texture and taste. In recent years, there has been a growing preference for natural foods free from additives, and the presence of these substances in ingredient lists has become a significant factor for consumers when selecting food. Consequently, the transmission of natural strains is an effective strategy for preparing high-quality fermented milk by improving its texture, enhancing its flavor, and increasing its biological activity. Thus, the quest for natural starter cultures has become a crucial aspect of the development of new natural fermented dairy products.

Thus, the objective of this study was to leverage the benefits of fermented mare’s milk and fermented cow’s milk to create a novel natural, additive-free fermented cow milk product that boasts a stable fermentation system, functional properties, and exhibits favorable gel characteristics. We inoculated various fermented mare’s milk added to sterile cow’s milk, compared the microbiota diversity among the different fermented mare’s milks, and comprehensively studied the characteristics of the resulting fermented cow’s milk. Water retention, rheology, texture, pH, and titratable acidity were analyzed to investigate the quality characteristics of gel properties of the fermented cow’s milk. It is important that we associate these properties with the strains in detail. Finally, this article provides a reliable strain system and theoretical basis for the development of new fermented cow’s milk products.

## 2. Materials and Methods

### 2.1. Materials

Fermented mare’s milk was sourced from 7 different vendors in different places. Sterile skimmed cow’s milk is a brand of Oldenburger, purchased from JD.com. All chemical reagents used were of reagent grade.

### 2.2. Sample Preparation

The fermented mare’s milk samples were labeled K1, K2, K3, K4, K5, K6, and K7. The back-slopping method was used to produce the fermented cow’s milk [12]. Four percent fermented mare’s milk was inoculated into sterile skim cow’s milk and activated passage was performed, with the samples being numbered accordingly. The specific procedures were as follows. The centrifuge tube, pipette tip, pipette tip box, and other experimental equipment were sterilized at 121 °C for 15 min. Then, 0.8 mL of fermented mare’s milk was inoculated into 20 mL of sterile skim cow’s milk at temperatures ranging from 40 °C to 45 °C with relative humidity of 85–95%. The samples, labeled Y1, Y2, Y3, Y4, Y5, Y6, and Y7, were then fermented at 42 °C for 12 h, followed by post-fermentation at 4 °C for 24 h. All samples were maintained in the same conditions, and the fermentation jars were kept tightly sealed.

### 2.3. Microbiota Diversity Detection

Detection of microbial diversity in all samples was conducted according to a previous method with a minor modification [13] with genomic DNA from various samples following the instructions provided in the corresponding DNA extraction kit (FINDROP, Guangzhou, China). Subsequently, DNA integrity and purity were assessed through 1% agarose gel electrophoresis and DNA concentration and purity determined using a NanoDrop One instrument (Thermo Fisher Scientific, Waltham, MA, USA). Utilizing the genomic DNA as a template, PCR amplification was conducted with barcoded primers and Premix Taq (TaKaRa, Tokyo, Japan), tailored to the selected sequenced regions. The concentration of PCR products was analyzed using GeneTools Analysis software (version 4.03.05.0, SynGene, Bengaluru, India). The PCR products were then mixed accordingly. The PCR mixture was purified using an E.Z.N.A.^®^ gel extraction kit (Omega, Fairfax, VA, USA) and eluted with TE buffer to recover the target DNA fragment. Subsequently, a NEBNext^®^ Ultra™ DNA Library Prep Kit (FINDROP, Guangzhou, China) for Illumina^®^ (Guangdong Magigene Biotechnology Co., Ltd., Guangzhou, China)was utilized to construct the library following the standard protocol, after which high-throughput sequencing was performed using the Hiseq or Miseq platforms. The original image data files obtained from sequencing were converted into raw reads via base calling analysis. The results were stored in FASTQ (fq) file format, which contained the sequence information of the reads and their corresponding sequencing quality information.

### 2.4. Determination of Water-Holding Capacity

The water-holding capacity of all the fermented cow’s milk was analyzed according to the paper [14]. A suitable quantity of fermented cow’s milk samples was accurately weighed using a precision balance (XS204, Mettler Toledo, Shanghai, China) and transferred to centrifuge tubes. Subsequently, the samples were centrifuged at 3500 r/min for 15 min using a refrigerated centrifuge (5424R, Eppendorf, Shanghai, China) to remove the supernatant. The weight of the sediment was then accurately measured, and the holding power was calculated using the following formula:

Holding capacity (%) = m_2_/m_1_ × 100%

where m_2_ is dry weight and m_1_ is wet weight.

### 2.5. Rheological Property Measurement

The fermented cow’s milk samples were tested using a rotating rheometer (KNX2212, Malvern Instruments Limited, Worcestershire, UK) as described by Ayyash et al. [15]. A stainless-steel flat probe with a diameter of 40 mm was used to ensure that the board control temperature was maintained at 25 ± 0.5 °C. The gap between the probe plane and the carrier mesa was adjusted to 1 mm. The storage modulus (G’) and loss modulus (G”) of fermented cow’s milk samples were measured using a dynamic frequency scanning technique. The frequency scanning range was set to 0.1–10 Hz. In the range of 0.1 to 1000 s^−1^, the shear scanning rate was obtained by logarithmic scanning, and a shear rate curve was drawn. Three parallel samples were measured for each treatment.

### 2.6. Determination of Texture Properties

TPA (total texture profile analysis) was conducted using a dairy quality tester from Chao Qi Technology Development Co., Ltd., based in Beijing, China. The texture of fermented cow’s milk samples was determined according to the paper with a little modification [16]. Initially, height calibration was performed with an empty fermenter using a probe model TA/BE set to a height of 60 mm. The speed was adjusted to 20 mm/s. Following this, seven samples were tested, with each sample undergoing three repetitions.

### 2.7. pH Value and Titratable Acidity Measurement

The titratable acidity and pH of each sample were determined using the method reported by Wang [17]. The pH was determined using a pH meter (FE28, Mettler Toledo, Shanghai, China). The device was utilized to record the pH values of seven different fermented cow’s milks, with each sample being tested three times. The acidity of the titration was measured by an automatic potentiometric titrator (T5, Mettler Toledo, Shanghai, China). Specifically, approximately 5 mL of the sample was taken and diluted with 20 mL of sterile water. Subsequently, a 0.1 mol/L NaOH solution was prepared. A measuring cup containing the fermented cow’s milk diluent was placed on the agitator and the NaOH was connected. Stirring was initiated after connecting the pH meter and continued until the pH reached 8.2. The volume of NaOH consumed was then recorded.

### 2.8. Statistical Analysis

The experiments were conducted with three replicates. All trial data are expressed as means ± standard deviation (X ± SD) and were processed using IBM SPSS Statistics 27 software. Univariate analysis of variance was utilized to analyze the differences, with *p* < 0.05 serving as the criterion for significance. The results were analyzed and visualized with Origin Pro 2024 or GraphPad Prism 10 software.

## 3. Results and Discussion

### 3.1. Microbiota Analysis and Functional Prediction Analysis

**Figure 1:**
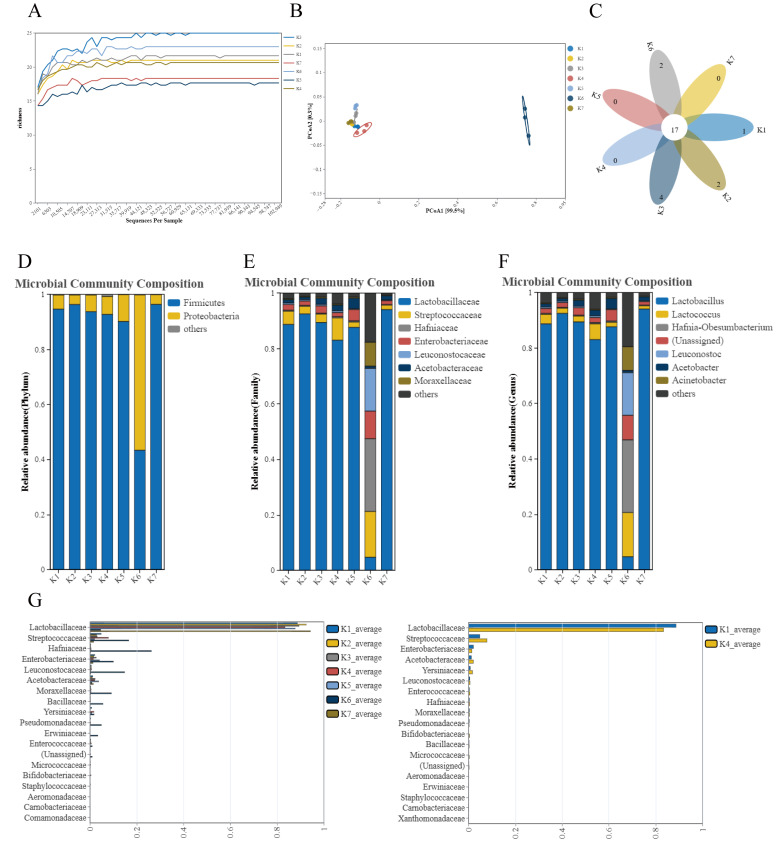
Microbiota analysis of different fermented mare’s milk: (**A**) α-diversity analysis, (**B**) β-diversity analysis, (**C**) number of OTUs in different groups, (**D**) relative abundance of bacteria at the gate level, (**E**) relative abundance of bacteria at the family level, (**F**) relative abundance of bacteria at the genus level, and (**G**) distribution of comparative microbiota.

The results of α-diversity analysis are displayed in Figure 1A. As indicated, the sequencing data in this test were sufficient to reflect the vast majority of microbial diversity information within the sample [18]. The species richness of K3 was the highest and that of K5 the lowest among the seven samples. In Figure 1B, the β-diversity analysis of PCoA indicates that samples with closer spatial distances exhibit a more similar species composition structure. It can be concluded that the species composition of K6 has the least similarity to the other components, while the others exhibit good similarity. K1 and K4 are the most similar among the different samples. Figure 1C displays the number of OTUs shared among multiple samples and the number of OTUs unique to each sample, directly reflecting the overlap of OTUs between the samples [19]. As illustrated, all samples share 17 identical operational taxonomic units (OTUs), while the non-overlapping portion represents the unique OTUs found in the seven different types of fermented mare’s milk. Samples K4, K5, and K7 do not have any specific OTUs. Subsequently, the relative abundance of each taxon is presented, revealing the variations in the abundance and relative abundance of bacteria present in different types of sour mare’s milk. As shown in Figure 1D, at the gate level, except for sample K6, *Proteobacteria* exhibits a higher relative abundance than *Firmicutes*. The relative abundance of *Firmicutes* in the other six groups was higher than that of *Proteobacteria*. This may be the reason for the difference between K6 and the other samples. Figure 1E illustrates that at the family level, the relative abundance of *Lactobacillaceae* in sample K6 is notably low, whereas the relative abundance of *Lactobacillaceae* in the other six groups is high. Additionally, the relative abundance of *Streptococcaceae*, *Hafniaceae*, *Enterobacteriaceae*, *Leuconostocaceae*, and *Moraxellaceae* in K6 was significantly higher than in the other six groups. Figure 1F indicates that at the genus level, the relative abundance of *Lactobacillus* in sample K6 is significantly low, whereas the relative abundance of *Lactobacillus* in samples from the other six groups is high. Additionally, the relative abundance of *Lactococcus*, *Hafnia-Obesumbacterium*, *Leuconostoc*, *Acetobacter*, and *Acinetobacter* in K6 is considerably higher than in the other six groups. Figure 1G illustrates the distribution of comparative microbiota within the sample group. All species of sour horse milk, except for K6, were predominantly composed of *Lactobacillaceae*, with K7 exhibiting the highest relative abundance. K6, on the other hand, contained *Streptococcaceae*, *Hafniaceae*, *Enterobacteriaceae*, *Leuconostocaceae*, *Moraxellaceae*, *Bacillaceae*, *Pseudomonadaceae*, and *Erwiniaceae.*

Typically, the strains found in commercially available fermented cow’s milk are *Lactobacillus delbrueckii* subsp*. bulgaricus* and *Streptococcus thermophilus*, which contribute to the stability and recognition of the fermented cow’s milk microbiota [20]. In this study, from a microbiota perspective, it is anticipated that fermented cow’s milk can be transformed from functional fermented mare’s milk, making it more appealing to consumers [21]. The fermented cow’s milk strains developed by researchers include *Limosilactobacillus fermentum*, *Lactiplantibacillus plantarum*, *Bifidobacterium animalis* subsp*. Lactis*, *and Lactococcus lactis* subsp*. lactis*, among others [22]. It is believed that the combined effect of all the bacteria present in fermented cow’s milk will enhance the functionality of fermented mare’s milk, rather than relying on single-strain fermentation alone [22]. Therefore, we continue to evaluate the grafted fermented cow’s milk.

**Figure 2:**
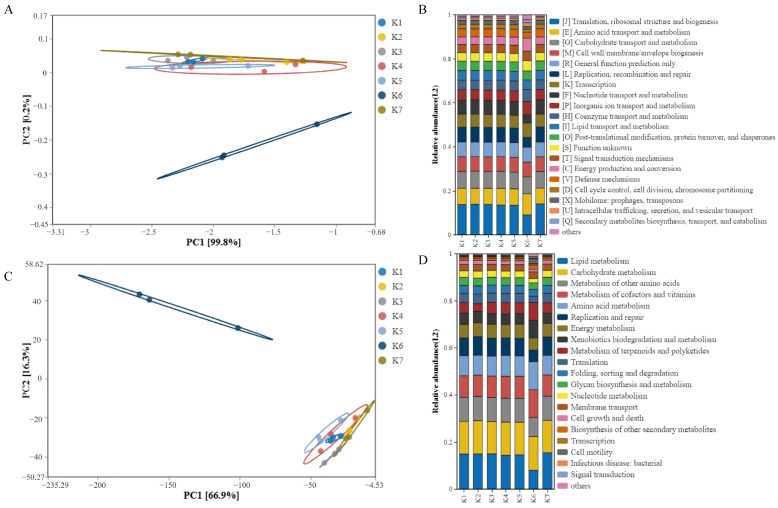
Functional prediction analysis. (**A**) PCA from COG functional analysis, (**B**) PCA from KEGG functional analysis, (**C**) functional prediction results from GO, and (**D**) functional prediction results from KEGG.

PCA from COG functional analysis and PCA from KEGG functional analysis are presented in Figure 2A and 2B, respectively. It is unsurprising that the functional prediction results for K6 are similar to those of the microbiota analysis, and it is comprehensible that the microbiota exhibits significant differences, with its functional prediction results also diverging from those of other samples. The PCA results of the other groups illustrate the similarities within each group. Apart from the K6 group, the strains were not significantly different at the genus level, and the functional prediction results did not indicate particularly noteworthy differences in either GO or KEGG (Figure 2C,D).

### 3.2. Description of Fermented Cow’s Milk Sample

**Figure 3:**
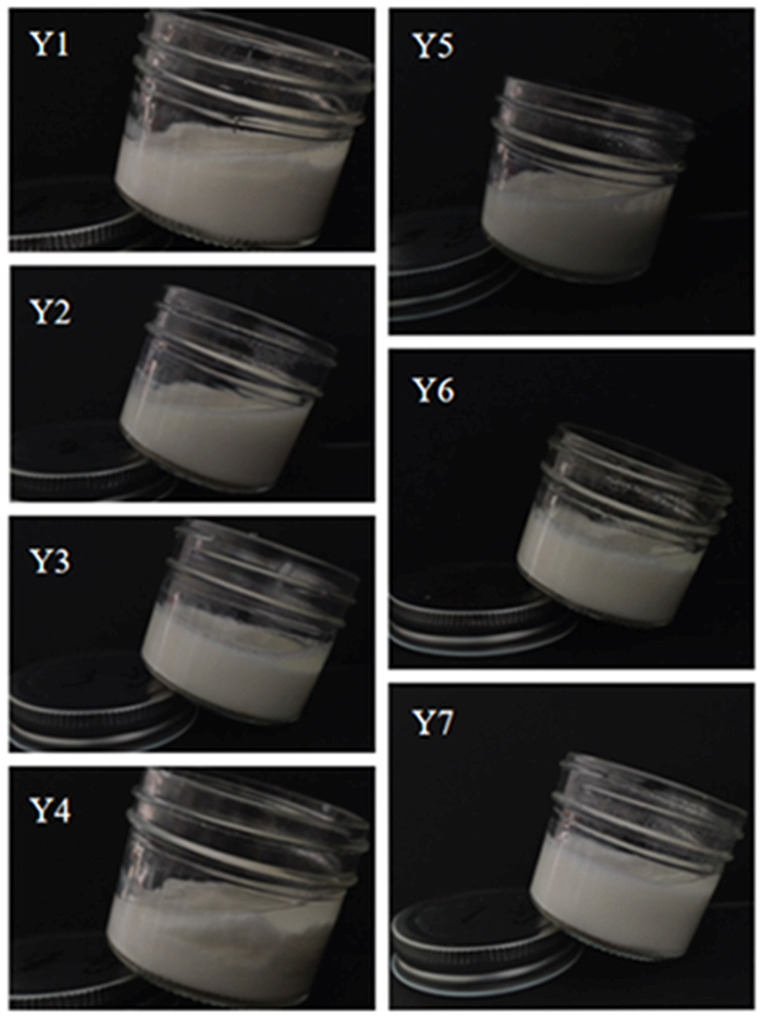
Photographs of inoculated fermented cow’s milk samples after inoculation with fermented mare’s milk to show differences in coagulation.

Seven types of fermented mare’s milk samples, each with a concentration of 4%, were inoculated into defatted pure milk for fermentation. Pictures of the fermented cow’s milk samples are depicted in Figure 3. Specifically, the solidification degree of Y7 is weak. Fermented cow’s milk is a popular fermented dairy product, and its desired texture is a result of the appropriate solidification process, which is closely related to the fermentation strains. Additionally, the low diversity of Y7 bacteria may also deteriorate their texture, and samples with low α diversity exhibit poor texture. Alpha diversity is positively correlated with texture parameters such as hardness and cohesion. Diverse microbiota communities offer a range of metabolic functions and interactions that are crucial for normal fermentation and the production of texture-related metabolites. Conversely, low alpha diversity can restrict metabolism and disrupt the fermentation process, thereby affecting the texture of fermented cow’s milk. The superior texture of Y3 fermented cow’s milk is due to the specific relative abundance of multiple bacterial families. *Leuconostocaceae*, known for their role in lactic acid fermentation [23], are low in Y3, which prevents over-acidification and the associated rough texture. *Lactobacillaceae*, which are highly abundant, produce EPS to enhance viscosity and texture [24], so the texture of Y3 and Y6 was relatively viscous, which may be due to the high abundance of *Lactobacillaceae* in K3 and K6. The K4 fermented mare’s milk sample had a relatively low abundance of *Lactobacillaceae* and a high abundance of *Acetobacteraceae*, the producers of acetic acid that can disrupt the texture, which may be the reason why Y4 had a weak texture. The content of *Streptococcaceae* is low, but stable. In particular, the EPS secreted by *Streptococcus thermophilus* can improve the rheological properties of yogurt and is widely used in commercial yogurt [25].

In fermented cow’s milk, the relative abundance of *Moraxellaceae*, *Leuconostocaceae*, and *Acetobacteriaceae* was extremely low and the sample differences were minimal. Because of the natural fermentation of fermented mare’s milk, the composition of the strains was inconsistent, especially in sample K6. *Moraxellaceae* was a non-fermenting bacterium in the fermentation system and can disrupt the normal metabolism of fermenting bacteria by competing for limited nutrients such as sugar and amino acids. Its relative abundance was higher in sample K6 compared to other samples. However, other genera in sample K6 had relatively high abundance, especially *Lactobacillus*, *Lactococcus*, *Leuconostoc*, and *Acetobacter*, which all had beneficial effects on the fermentation of milk. *Acetobacter* can generate acetic acid by metabolizing lactose or ethanol in the symbiotic fermentation system, regulate the pH value of the system, promote the proliferation of *Lactobacillus*, accelerate the gelation of casein, and thereby enhance the viscosity and texture stability of yogurt [26]. In addition, certain strains of *Leuconostoc* produce exopolysaccharides that increase viscosity and gel strength, enhance water retention and taste, and their metabolites also influence flavor [23]. In K6 samples, their relative abundance was high, which may be one of the reasons for the improved texture of the Y6 fermented cow’s milk. In contrast, the low abundance of *Leuconostoviaceae* in other samples is due to its slow growth rate, weak competitive ability for nutrients, and an incomplete environment [27]. The relative abundance of *Lactobacillaceae*, *Enterobacteriaceae*, and *Streptococcaceae* is similarly low, with minimal variation in their population sizes [28]. However, the relative abundance of *Lactobacillaceae* is relatively high. When combined with appropriate amounts of other bacteria, this microbial composition contributes to the excellent texture of fermented cow’s milk [29], with *Lactobacillaceae* playing a particularly critical role in promoting protein coagulation and the production of extracellular polysaccharides, which enhance the hardness, viscosity, and smoothness of fermented cow’s milk [24]. Moreover, it is speculated that *Lactobacillaceae* or other acid-producing bacteria may dominate the microbial system in fermented strains, secreting a significant amount of acid that leads to casein precipitation. The presence of other strains could be inhibited due to the influence of metabolic products, such as acid, altering their living environment. Consequently, different samples with distinct strains may exhibit similar physicochemical properties. As such, it is evident that strains from different fermented mare’s milk varieties influenced the solidification degree of the fermented cow’s milk samples. The variation in strains also dictated the texture of the fermented cow’s milk samples.

### 3.3. pH and Titratable Acidity Analysis

**Figure 4:**
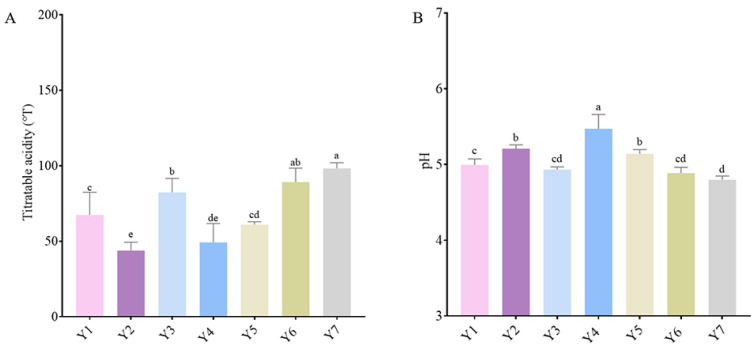
Analysis of pH and titratable acidity. (**A**) Titratable acidity of the seven samples, and (**B**) pH values of fermented cow’s milk samples. Different letters indicate significant differences between groups (*p* < 0.05). The same letter indicates no significant difference (*p* > 0.05).

As depicted in Figure 4, different fermented cow’s milk samples exhibit varying pH levels. Figure 4A illustrates the titratable acidity of the seven samples. Samples Y2, Y4, and Y5 required less NaOH volume, whereas samples Y1, Y3, Y6, and Y7 consumed more NaOH. In Figure 4B, it is evident that the pH values of fermented cow’s milk samples Y1, Y3, Y6, and Y7 are all below 5.0, with Y7 having the lowest pH value. Additionally, Y7 has the highest relative abundance of *Lactobacillaceae*, indicating that this family produces a significant amount of acid. From a metabolic pathway perspective, most members of the *Lactobacillaceae* family utilize glycolysis to convert sugars into pyruvate. Subsequently, pyruvate is converted into lactic acid by lactate dehydrogenase, which is the primary process of acid production [30]. Additionally, some bacteria are capable of heterolactic fermentation, producing acidic substances such as acetic acid in addition to lactic acid, thereby enhancing the overall acid production [30]. Regarding physiological characteristics, the cell membrane of *Lactobacillaceae* possesses a unique proton transport system. This system efficiently expels hydrogen ions generated within the cell, maintaining cellular acid–base balance and ensuring continuous acid-producing metabolism [31]. Similarly, the Y6 sample exhibited superior performance in terms of titratable acidity and pH, leading us to conclude that *Hafniaceae*, *Enterobacteriaceae*, *Leuconostocaceae*, *Moraxellaceae*, *Bacillaceae*, *Pseudomonadaceae*, and *Erwiniaceae* may have contributed to this outcome. These families play a crucial role in enhancing the acidity of fermented cow’s milk [32]. *Leuconostocaceae* also produce other organic acids while producing lactic acid. *Bacillaceae* enzymes catalyze the conversion of sugars into organic acids [33]. *Pseudomonadaceae* exhibit strong adaptability and can sustain metabolic acid production in a fermentation environment [34].

### 3.4. Water-Retention Analysis

**Figure 5:**
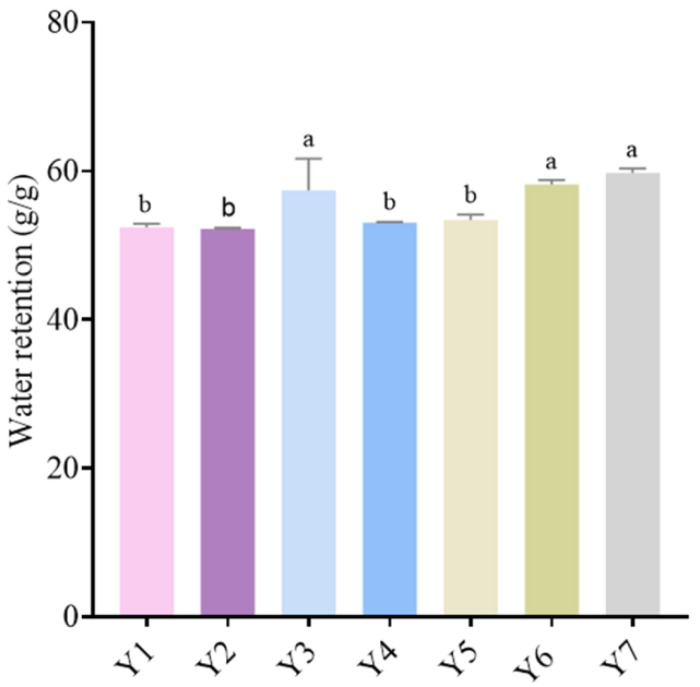
Water-retention analysis of the fermented cow’s milk samples. Different letters indicate significant differences between groups (*p* < 0.05). The same letter indicates no significant difference (*p* > 0.05).

Figure 5 illustrates the water-retention ratio of fermented cow’s milk samples Y1 to Y7. In the figure, it is evident that among the seven fermented cow’s milk samples, Y3, Y6, and Y7 exhibit a significantly (*p <* 0.05) stronger water-retention capacity than the other groups. *Lactobacillus*, a member of the family *Lactobacillaceae*, plays a key role in enhancing the water retention of fermented cow’s milk [35]. During fermentation, these bacteria convert lactose into lactic acid, which results in lower pH. The change in pH value causes milk proteins, particularly casein, to denature and aggregate, forming a complex three-dimensional protein network [36]. Because water molecules are physically trapped within the pores of this protein network, the water retention of fermented cow’s milk is enhanced. Additionally, the metabolic by-products of *Lactobacillaceae* can interact with water molecules and the protein matrix, further enhancing the water-holding capacity of yogurt. As shown in Figure 5, Y7 has the lowest α diversity [37].

Low α diversity in the yogurt microbiota, which measures the richness and uniformity of species within a single sample, enhances its stability. In fermented cow’s milk, the low α diversity indicates that certain beneficial strains (such as *Lactobacillaceae*) predominate. They efficiently ferment lactose to produce lactic acid, which significantly lowers the pH. The acidic environment promotes the denaturation and aggregation of milk proteins, forming a well-structured three-dimensional protein network. This network more effectively traps water molecules, thereby enhancing the water-retention capacity of fermented cow’s milk. Thus, we can speculate that it is primarily the *Lactobacillaceae* that play a role.

### 3.5. Rheological Analysis

**Figure 6:**
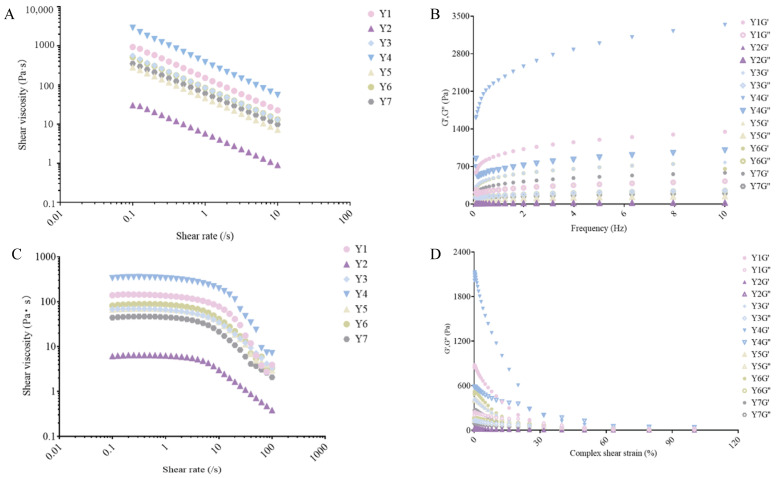
Rheological analysis of the fermented cow’s milk samples.

Shear rate, frequency, and amplitude tests were conducted on fermented cow’s milk samples Y1, Y2, Y3, Y4, Y5, Y6, and Y7, with the results presented in Figure 6. Apparent viscosity serves as a rheological index of inherent physical properties, indicating the structure of fermented cow’s milk and the pairwise motion of the milky liquid phase [38]. As depicted in Figure 6A,C, the apparent viscosity of all fermented cow’s milk samples decreased with an increase in shear rate. Consequently, fermented cow’s milk exhibits shear-thinning behavior and is classified as a pseudoplastic fluid [39]. As illustrated in Figure 6A, among the Y1 to Y7 samples, Y1 and Y4 exhibit the highest apparent viscosity, while the Y2 sample has the lowest. Figure 6B shows that the elastic modulus (G’) and viscous modulus (G”) of all samples increase with frequency. Moreover, the elastic modulus (G’) of all samples remains greater than the viscous modulus (G”), suggesting that the fermented cow’s milk system predominantly displays elastic behavior [39]. It is known that fermented cow’s milk has a semisolid, weak gel structure. Among all samples from Y1 to Y7, it can be observed that the elasticity of Y4 is superior to that of the others: as seen in Figure 6C, the viscosity of both Y1 and Y4 is relatively high. As illustrated in Figure 6D, with the rise in complex shear strain, the elastic modulus (G’) and viscous modulus (G”) of all samples decreased, with the elastic modulus (G’) consistently exceeding the viscous modulus (G”). This suggests that the fermented cow’s milk system predominantly exhibits elastic behavior. Considering the experimental results above, the increased viscosity and improved texture of fermented cow’s milk may be attributed to the interaction between *Lactobacillaceae* and *Streptococcaceae*. During the fermentation process, these two bacterial families work in concert to enhance the viscosity of the product. *Lactobacillus bulgaricus*, a member of the *Lactobacillaceae* family, produces exopolysaccharides that increase intermolecular interactions, thereby improving viscosity. Additionally, it ferments lactose to produce lactic acid, which alters the pH value and affects protein structure and interaction, thus indirectly influencing the viscosity of the fermented cow’s milk [40]. *Streptococcus thermophilus* and *Lactobacillus* cooperate, with their metabolites promoting the growth of *Lactobacillus*. Additionally, their metabolites and enzymes synergistically act on milk components to facilitate protein aggregation and cross-linking [41]. Simultaneously, proteases secreted by *Streptococcus* decompose casein, and the resulting products form a complex network structure with polysaccharides, further enhancing the viscosity of fermented cow’s milk [42].

### 3.6. Texture Characteristic Analysis

**Figure 7:**
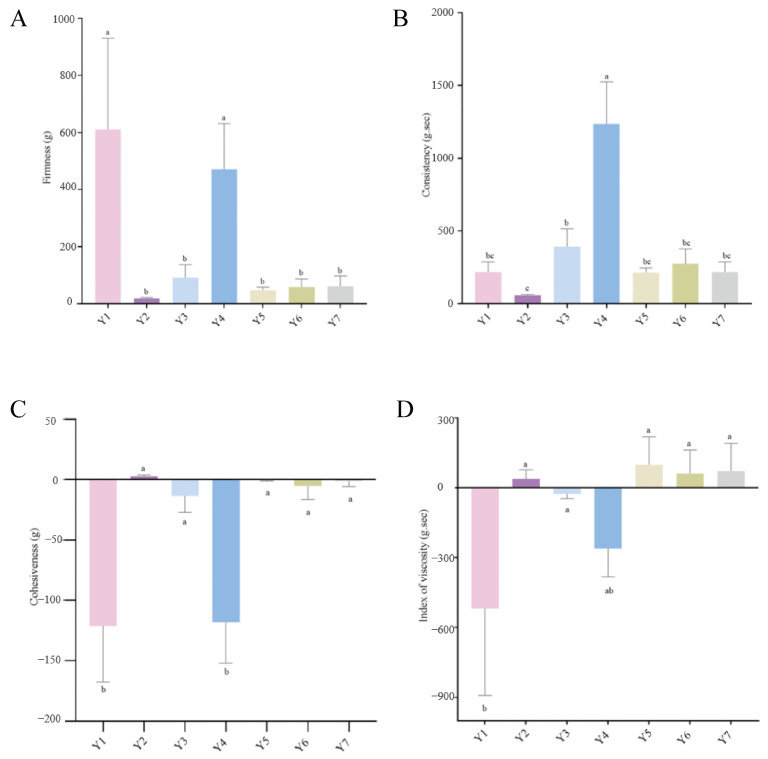
Texture characteristic analysis of the fermented cow’s milk samples. (**A**) Firmness, (**B**) Consistency, (**C**) Cohesiveness, and (**D**) Index o fviscosity. Different letters indicate significant differences between groups (*p* < 0.05). The same letter indicates no significant difference (*p* > 0.05).

The firmness, consistency, cohesiveness, and viscosity index of samples Y1, Y2, Y3, Y4, Y5, Y6, and Y7 are presented in Figure 7. Figure 7A indicates that Y1 and Y4 exhibit high firmness, whereas the other samples display relatively lower firmness. As illustrated in the figure, Y1 and Y4 exhibit the highest relative abundance of *Lactobacillaceae*, which is combined with other bacteria. However, when compared to other groups, the relative abundance of *Lactobacillaceae* is relatively low, potentially leading to increased sample hardness. The relative abundance of *Lactobacillaceae* is negatively correlated with the hardness of fermented cow’s milk. The low abundance of *Lactobacillaceae* in this fermented cow’s milk leads to increased hardness. This suggests that *Lactobacillaceae* produces lactic acid during fermentation, which promotes protein coagulation. A lower relative abundance of *Lactobacillaceae* results in less lactic acid, thereby increasing hardness. Additionally, interactions with other bacteria also affect the texture. Fermented cow’s milk is a semisolid food with liquid-like properties [43]. The fluidity of fermented cow’s milk reflects its consistency [44]. After inoculation with different strains, the consistency of fermented cow’s milk varied significantly (*p* < 0.05). The closer the consistency value is to zero, the weaker the system’s consistency becomes [45]. Figure 7B indicates that Y4 has the highest consistency among all samples, resulting in a denser fermented cow’s milk with poor fluidity. It is evident from Figure 1E that Y4 has a high concentration of *Lactobacillaceae* and numerous other strains. A large number of *Lactobacillaceae* in fermented cow’s milk can produce excessive lactic acid, potentially destroying the gel structure. Conversely, a significant presence of *Streptococcaceae* may enhance the production of protein cross-linking or exopolysaccharides (EPS), thereby increasing viscosity. Additionally, the diversity of strains can lead to synergistic effects [46]. The cohesiveness of fermented cow’s milk reflects the strength of the internal bonds within the protein network structure and indicates the degree of aggregation within the gel of the fermented cow’s milk [47]. The higher the bond value, the stronger the internal bond of the gel [48]. Given that the negative mean values of the adhesion and viscosity index suggest that the fermented cow’s milk sample adheres to the probe, the absolute values of the data will be utilized to compare the adhesion and viscosity index of the sample. As depicted in Figure 7C, the absolute values of Y1 and Y4 are high, indicating that the adhesion values of the Y1 and Y4 samples are high, the gel diffusion rate is slow, and the distance is long. This further suggests that the internal structure of these foods is difficult to decompose [48]. This result may be attributed to the relatively high abundance of Y1 and Y4 *Lactobacillaceae*. In comparison to other groups of *Lactobacillaceae*, the relative abundance of these bacteria is lower. In fermented cow’s milk, the relative abundance of *Lactobacillaceae* is typically high, playing a key role in the fermentation process [24]. They convert lactose into metabolites, such as lactic acid, which lowers the pH (acidity) and causes milk proteins to coagulate and gel, giving fermented cow’s milk its distinctive texture. When the relative abundance of *Lactobacillaceae* in fermented cow’s milk is lower than in other groups, this may indicate different microbial populations of species involved in fermentation and gelling is insufficient. This can lead to inadequate fermentation, affecting the solidification and gelling process of proteins, resulting in fermented cow’s milk that is overly viscous with a slow gel diffusion rate over a long distance. At the microscopic level, the structure of the protein network inside fermented cow’s milk is imperfect and unstable and the interaction between molecules is weak, making the internal structure of the food difficult to decompose. In Figure 7D, the higher the viscosity index, the more viscous the texture. From the results of samples Y1 to Y7, it is evident that Y1 and Y4 samples have a higher viscosity index and are more viscous in comparison. This may also be related to the abundance of *Lactobacillaceae*.

## 4. Conclusions

In this study, the microbiota from naturally fermented mare’s milk were introduced into skimmed milk for fermentation. A systematic comparison of the microbial structures in seven different milk fermentation systems was conducted. The physicochemical properties of the fermented milk and their rheological characteristics as starters were evaluated. The results indicated that the physicochemical properties of the fermented milk may not necessarily correlate with the diversity of the microbiota. Notably, the Y1 and Y4 systems, which utilized mixed bacterial fermentation, demonstrated superior fermentation performance. The characteristic microbial communities in these systems included *Lactobacillus*, *Lactococcus*, *Hafnia-Obesumbacterium*, *Leuconostoc*, *Acetobacter*, and *Acinetobacter.* The study revealed that the genera *Lactococcus and Lactobacillus* might play an essential role in the milk fermentation process. *Lactobacillus* produced substantial amounts of acid to lower the pH value and enhance the water-holding capacity of fermented yogurt. Moreover, the synergistic effect between *Lactobacillaceae* and *Streptococcaceae* was closely associated with increased viscosity and improved texture of the fermented milk. These findings provide innovative methodological support and a theoretical basis for developing new fermented dairy products and screening suitable fermentation strains.

## Data Availability

The original contributions presented in this study are included in the article. Further inquiries can be directed to the corresponding authors.
